# For the Greater Good? The Devastating Ripple Effects of the Covid-19 Crisis

**DOI:** 10.3389/fpsyg.2020.577740

**Published:** 2020-09-29

**Authors:** Michaéla C. Schippers

**Affiliations:** Department of Technology and Operations Management, Rotterdam School of Management, Erasmus University Rotterdam, Rotterdam, Netherlands

**Keywords:** Covid-19 crisis, disrupted supply chains, conspiracy theories, social influence, life crafting, increased inequalities, coping, agnotology

## Abstract

As the crisis around Covid-19 evolves, it becomes clear that there are numerous negative side-effects of the lockdown strategies implemented by many countries. Currently, more evidence becomes available that the lockdowns may have more negative effects than positive effects. For instance, many measures taken in a lockdown aimed at protecting human life may compromise the immune system, and purpose in life, especially of vulnerable groups. This leads to the paradoxical situation of compromising the immune system and physical and mental health of many people, including the ones we aim to protect. Also, it is expected that hundreds of millions of people will die from hunger and postponed medical treatments. Other side effects include financial insecurity of billions of people, physical and mental health problems, and increased inequalities. The economic and health repercussions of the crisis will be falling disproportionately on young workers, low-income families and women, and thus exacerbate existing inequalities. As the virus outbreak and media coverage spread fear and anxiety, superstition, cognitive dissonance reduction and conspiracy theories are ways to find meaning and reduce anxiety. These behavioral aspects may play a role in the continuance of lockdown decisions. Based on theories regarding agnotology (i.e., the ways ignorance or doubt about certain topics is created by means of withholding or presenting information in a certain way), social influence, superstition and stress and coping, I seek to explain the social and behavioral aspects of human behavior in times of crises. Both the Covid-19 crisis itself as well as the resulting economic and (mental) health crisis are global problems that may require global solutions. I present a model of drivers and outcomes of lockdown behaviors and offer suggestions and a tool to counteract the negative psychological effects by means of online life crafting therapeutic writing interventions.

## Introduction

‘A sad soul can kill you quicker than a germ’

– John Steinbeck in his novel Travels with Charley. In search of America (1962)

Humankind is currently facing an unprecedented global crisis. The decisions we make today will shape the worlds for years to come. As a massive public health campaign was launched aimed at slowing the spread of the COVID-19 virus, scholars have outlined social and behavioral evidence that help shape policies aimed at influencing human behavior such as social distancing, staying at home, and handwashing (Bavel et al., 2020). Many countries have taken lockdown measures to enforce this behavior (Ren, 2020). At the same time, it now becomes clear that many of the measures taken, are causing an immense humanitarian disaster and the cure seems to be much worse than the disease (Rojas et al., 2020; Zhang et al., 2020). The myopic attendance to Covid-19/SARS-CoV-2^1^ and disease control, has led to many consequences that affect people’s mental and physical health and safety (Holmes et al., 2020). For instance, early on in the crisis it was already estimated that at least 100 million people will die from hunger due to the lockdowns (Zetzsche, 2020), and as the lockdowns continue or even renewed in the upcoming times, the costs in terms the socio economics could be very high.

Several authors have commented on the harms of non-evidence-based measures that many countries have taken, on the basis of failed predictions on the severity of the problem (Ioannidis, 2020; Ioannidis et al., 2020). This has led some authors to suggest that agnotology, or the culturally induced ignorance or doubt, particularly the publication of inaccurate or misleading scientific data, might be at play in the current crisis with respect to the side effects of the lockdowns (Lee, 2020). Agnotology is described as more than just ignorance or the absence of knowledge. It is often the outcome of cultural and political struggles (Proctor and Schiebinger, 2008). An historical example is the tobacco industry trying to hide the negative effects of smoking from the general public and shows “how institutions and individuals work hard to confuse and cloud any evidence that might show us what is actually happening in particular places” (Slater, 2019, p. 24). According to Srivatsa and Stewart (2020): “Epidemic response strategies typically involve infection control, health systems strengthening, and other disease containment strategies. However, intense focus on pathogen transmission can lead responders to overlook trauma and psychosocial damage to individuals and communities during and following an epidemic.” Indeed, Brooks et al. (2020) showed that in previous, more localized lockdowns for related viruses the psychological damage was quite severe, and they conclude that “the potential benefits of mandatory mass quarantine need to be weighed carefully against the possible psychological costs.” (Brooks et al., 2020, p. 912). In addition to psychological costs, other negative consequences stem from the fact that many medical procedures have been postponed, and from people staying away from medical care out of fear from Covid-19. Consequently, the toll on non-Covid patients will be much greater than Covid deaths (Maringe et al., 2020; Rosenbaum, 2020). Moreover, as many businesses are closed and supply chains blocked, the socio-economic effects are beyond comprehension (cf. Fernandes, 2020; Ivanov, 2020). As many countries are in some form of lockdown, or just coming out of a lockdown, it becomes clear that this has negative side effects for the general population, in terms of mental and physical health, as well as on the economic side (Ren, 2020; VanderWeele, 2020; See Table 1 for a non-exhaustic overview of side and ripple effects). Although the consequences of the lockdowns are currently hard to assess fully as the situation is still unfolding, and some countries may decide to renew lockdowns in the upcoming time, the severity of these ripple effects can hardly be overestimated at this point in time.

**TABLE 1:** Non-exhaustive overview of the side and ripple effects of the pandemic and related lockdown measures, including references.

**Physical Health**	
**Side Effect**	**References**
Estimated 100 million casualties in low and middle-income countries, as an indirect effect of the virus, and the lockdown measures (early estimate).	Zetzsche, 2020
138 million people face starvation as economies and livelihoods are interrupted by the pandemic (updated estimate).	Kennedy et al., 2020
COVID-19 likely to lead to increased maternal and child mortality indirectly, via disrupted healthcare, decreased food access, health system and economic collapse.	Roberton et al., 2020
Access to other forms of healthcare may be limited, as doctors are redirected, and people fear seeking care, leading to worse health outcomes in the long run. Risk of many deaths from health problems not related to covid-19.	[Bibr B51][Bibr B45]
There has been a significant increase in the number of major amputations during lockdown as patients wait longer to seek medical care for non-covid-19 illnesses.	Schuivens et al., 2020
Quarantine stress increases the risk of cardiovascular health problems.	Mattioli et al., 2020
Access to reproductive healthcare during lockdowns is limited which leaves some women without access to care they need.	[Bibr B117][Bibr B74]
Global condom shortage may be looming as manufacturing is shut down, which is likely to result in increase in sexually transmitted infections and unplanned pregnancies, especially in poorer countries.	Chin, 2020
**Mental Health**	
The pandemic could lead to a significant rise in suicide mortality in the coming months.	[Bibr B95][Bibr B84]
Worsening mental health concerns as stress, depression, and anxiety increases.	Fiorillo and Gorwood, 2020
Current decrease in access to mental healthcare may result in worsening mental health of the general population, with people with existing conditions being most at risk.	Torales et al., 2020
Those with pre-existing mental health conditions are most at risk of having increased mental health issues due to the pandemic.	Druss, 2020
Pandemic triples anxiety and depression symptoms in new mothers.	Davenport et al., 2020
A significant increase in rates of insomnia may worsen stress, anxiety, and other existing mental health issues, especially in frontline workers.	[Bibr B91][Bibr B101]
Mandatory lockdowns or quarantines may have an especially large negative effect on individuals suffering from social anxiety.	Zheng et al., 2020
Economic Effects	
The total worldwide economic cost of the pandemic could reach $8.8 trillion.	Takagawa, 2020
The pandemic coupled with government relief packages being put into place could result in a worldwide deficit of $30 trillion by 2030.	Assi et al., 2020
Half of world’s workers ‘at immediate risk of losing livelihood due to coronavirus’.	Inman, 2020
Despite efforts to minimize layoffs, 60 million EU jobs are at risk, and mass layoffs are predicted for the near future.	[Bibr B119][Bibr B1]
Over 54 million Americans have applied for unemployment aid for the first time.	Jones C., 2020
The lockdown is likely to have a disproportionately large effect on young workers, who make up the majority of industries highly affected by layoffs (service industry etc.).	Kochhar, 2020
Social Effects	
The physical and mental health of frontline workers like healthcare workers, and those working in food distribution may be at risk.	[Bibr B71][Bibr B46]
Domestic violence deaths have more than doubled from this period in previous years.	[Bibr B47][Bibr B13]
Homeless and refuge population left at risk as lockdown limits access to help resources, and leaves them unable to shelter in place.	[Bibr B136][Bibr B89]
Increase in gun purchases and gun violence in the USA since the beginning of the pandemic.	Schleimer et al., 2020
The pandemic will likely result in an additional 30 years to close the gender pay gap in Britain.	Hunt, 2020
**Effects on Children**	
Unicef warns 1.2 million children could die malaria, pneumonia, and diarrhea during the lockdowns in developing countries.	Newey, 2020
The pandemic is likely to leave a lasting influence of the mental health of children and adolescents.	Fegert et al., 2020
368 Million children missing out on meals at school and school closures overly affects children from poorer communities.	[Bibr B26][Bibr B150]
Children from pooper communities likely to suffer the most as education moves online for many communities, and nearly half the world still doesn’t have ready access to the internet.	COVID-19’s Devastating Impact on Children, 2020

**FIGURE 1:**
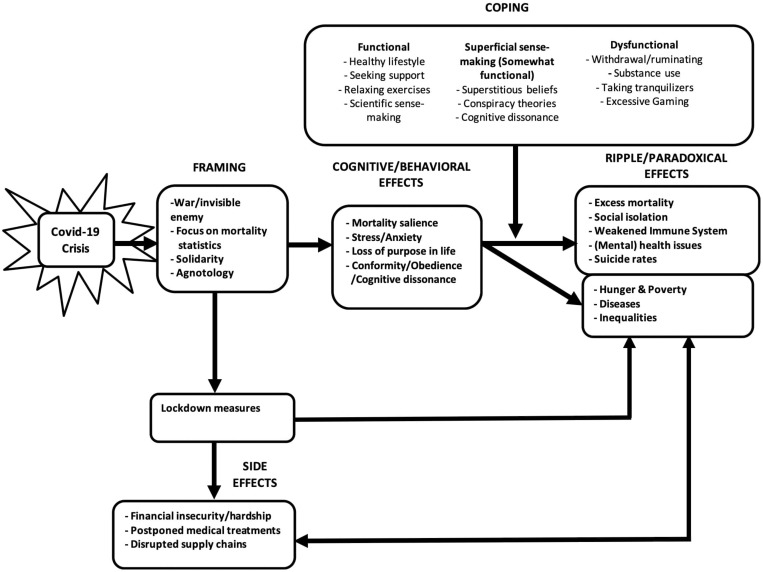
Theoretical model of the consequences of the Covid-19 crisis, including mediating and moderating variables.

In the current review, I aim to elucidate mechanisms that explain the attitudes and behaviors of people in general as well as behavioral mechanisms in the current situation (See Figure 1). I will describe the processes through which the decisions for the lockdowns in many countries are internalized and upheld through a process of framing, social influence and superstition. I will focus on the effects that the lockdowns have on the general population, rather than on the effects on individual patients and caregivers, which I deem to be a special group but that has been given attention elsewhere (Lin et al., 2007; Lee et al., 2018; Kim et al., 2019). I will describe how the framing of the situation by political leaders and in the popular press influences mortality salience, and stress and anxiety, and in turn drives cognition and behavior (i.e., cognitive dissonance, conformity and obedience). Many of the lockdown measures however are paradoxically related to a weakened immune system, stemming from a loss of purpose in life, social isolation and related mental health issues, leading to outcomes such as excess mortality, increased suicide rates, and an increase in non-Covid related diseases (cf. Torales et al., 2020; Zhou et al., 2020). Since these effects are stronger for vulnerable groups, this will widen the existing inequalities (Holmes et al., 2020). I will give attention to this paradox that, as a society, we seem to be compromising the immune system and economic security of the majority of people in the lockdown situation. The effects will in part be moderated by the effectiveness of the coping styles used by individuals (See Figure 1). Due to space constraints, I will give a brief summary of each topic, and also briefly describe how they are related and influence each other. In this review, I do by no means try to be exhaustive, but will limit myself to the main drivers of human behaviors, and the expected consequences. The model may act as recommendation for future research, as the model, although based on prior research, has not been tested yet. Since other researchers already suggested policy considerations in order to help decision-makers prevent the most horrifying scenario such as a scenario of excess mortality from extreme hunger and famine (e.g., Hevia and Pablo Andrés, 2020; Schippers and Martins Van Jaarsveld, 2020; Zetzsche, 2020), I will not repeat that here. Below. I start with the explaining that the way the situations is framed result I adherence to lockdown measures. Following I explain the right side of the model, the results and negative side effects, before discussing the remainder of the model (See Figure 1 and Table 1, supplementary material). I will end with recommendations for interventions that may be used to mitigate the negative effects of the lockdown on the general population.

## Framing of the Situation and Lockdown Measures

As half of the world is in some kind of lockdown, this is arguably the largest psychological experiment ever (van Hoof, 2020), with ripple effects on every aspect of human life (Bavel et al., 2020; Nicola et al., 2020). As the virus spreads, and the government and media stipulates this, so does the spread of fear. The way the crisis is framed may be key to how people’s behavior is shaped under lockdown conditions (Bavel et al., 2020). In general, people have a stronger tendency to act when a problem is framed as death-preventing (losses) than life-saving (gain) (Chou and Murnighan, 2013; Bavel et al., 2020; Schippers and Martins Van Jaarsveld, 2020). The groundwork for these kind of framing effects was laid by prospect theory, which suggests that the pain of losing is about twice as strong as gaining the same amount, and people are more motivated to avoid losses than to achieve gains. For instance, when a call for blood donations was described as death-preventing (losses), rather than life-saving (gains), and as being urgent, this boosted donations (Chou and Murnighan, 2013). In terms of goal framing, if a message is framed as avoiding negative consequences (loss frame), this will generally have a stronger impact on human behavior than when it is framed as having positive consequences (gain frame; Krishnamurthy et al., 2001). In the current situation, the focus is on death-prevention and on preventing from infection with the Corona virus, which can in part explain the sheer one-sided focus and news coverage on this perspective. Prior research has focused on the persuasive effectiveness of messages, especially for promoting health behaviors (Rothman et al., 1993; Levin et al., 1998), and willingness to sacrifice for the greater good (Bilandzic et al., 2017). This mechanism will also be sustained by mortality salience. Terror management theory postulates that people confronted with reminders of death increase striving to maintain faith in one’s cultural worldview, self-esteem, and attachment security (Pyszczynski et al., 2015). Research on social dilemmas (i.e., a conflict between immediate self-interest and longer-term collective interests), shows that under certain conditions people are more inclined to forego their own interests in the interest of the collective longer-term goal of survival. This research gives insight into the ways in which cooperation occurs (for a review see van Lange et al., 2013). As the situation is also oftentimes presented as a social dilemma, and even as a false dilemma (e.g., choice between security and health), this may amplify adherence to lockdown rules, despite the fact that many measures taken are not evidence-based (Ioannidis, 2020). The framing of the disease as a threat to humans, has made sure that most people adhere to the recommendations (Bavel et al., 2020). The way behavior is maintained is by social influence, forces that are often indirect, subtle and unconscious (Cialdini and Goldstein, 2004). Even so many countries have opted to regulate behavior by rules, regulations and enforcement. Besides, many media outlets have opted to present information in a biased manner, possibly in order to create a uniform narrative inducing people to follow guidelines issued by governments and health organizations.

## Results: Catastrophic Side Effects

The lockdown in many countries can have quite severe side effects on the physical and mental health of people (Brooks et al., 2020; for reviews see Jeong et al., 2016; Torales et al., 2020; Wang C. et al., 2020). The side effects so far seem to outweigh the positive effects and a recent historical overview of outbreaks concludes that: “History suggests that we are actually at much greater risk of exaggerated fears and misplaced priorities” (Jones D. S., 2020; p. 1683). The main side effects are: Excess mortality from causes other such as hunger, delayed health care, increase in effects mental health issues, suicide, increase in diseases such as measles, and increased inequalities due to school closures and job loss. These have ripple effects throughout society. In many countries emergency admissions, e.g., for cardiac chest pain and transient ischemic attacks, are decreased by about 50%, as people are avoiding hospital visits, which eventually will lead to higher death rates from other causes, such as heart attack and strokes (Sarner, 2020). Also, many medical treatments such as chemotherapy have not been given and were postponed (Sud et al., 2020). In terms of mental health effects, vulnerable groups, such as people with prior mental health issues might be at especially high risk (Jeong et al., 2016). Indeed, a survey by Young Minds revealed that up to 80% of young people with a history of mental health issues reported a worsening of their condition as a result of the pandemic and lockdown measures (Sarner, 2020). The mental health effects arguably affect the general population as a whole, and it has been suggested that this will be a global catastrophe (Izaguirre-Torres and Siche, 2020). During the lockdowns, mental health care is limited or not available at all, and the psychological effects can be devastating. Many people are likely to develop a wide range of mental health issues due to being quarantined, and/or as a result of job loss, such as low mood, insomnia, irritability, depression and posttraumatic stress disorder (Holmes et al., 2020; van Hoof, 2020). Not only is there fear and anxiety for oneself or loved ones becoming infected, there is also fear of financial hardship and uncertainty about what the future may bring. It is expected that there will be an enormous increase in hunger and poverty, in part due to distortions in many supply chains around the world (Boone et al., 2020; Buheji et al., 2020). This will be especially so in developing countries with prior challenges of socio-economic and livelihood issues (Buheji et al., 2020), which will more directly be related to excess mortality (Kalu, 2020; Zetzsche, 2020). Even so, the fact that the lockdowns have a lot of side-effects gets relatively little attention (Holmes et al., 2020), although some authors have recommended on when to release the lockdown (Layard et al., 2020). As some lockdowns endure or will be renewed in the upcoming time, the numbers and results presented here may prove to be quite conservative (cf. Mandel and Veetil, 2020), and public health initiatives are needed to reverse some of these devastating side effects (cf. de Jong et al., 2020; Guessoum et al., 2020; VanderWeele, 2020).

## Agnotology Induced Conformity, Obedience and Cognitive Dissonance

As the Covid-19 crisis had been framed as a “war against an invisible enemy” and the nurses and physicians are named ‘soldiers’ or ‘warriors’ in ‘the front line’ many biases and errors that humans tend to have, have become visible. During the crisis, media attention has been used to highlight information about the virus and spread of the virus, while being relatively ignorant to the fact that many measures have severe side effects such as hunger, job loss and increased inequalities. Governments and governmental institutions have been involved in making sure information is presented in a certain way, probably in an effort to ensure public commitment to the measures taken (Betsch et al., 2020). Moreover, this was often done in terms of false dilemma’s presenting the problem as a choice between for instance lives and livelihood (Prasad et al., 2020) and ignoring the fact that the costs of the measures fall on the global poor (Broadbent et al., 2020). In spite of this, the measures and framing have led not only to commitment to the measures, but also to agnotology by means of censorship, putting weight of facts differently as well as being inattentive to the severe side effects of the measures (Zhong et al., 2020).

The effects of framing on the extent to which people obey authorities, even if the orders given are against their better (moral) judgment has been under investigation for decades. Three famous experiments show the intricacies of social influence, which have become known as the Asch conformity experiment, the Milgram obedience experiment, and the Stanford Prison experiment. In the Asch experiment, it was shown that even in a very unambiguous situation, with one clear right answer, 75% of people could be persuaded to give the wrong answer as long as the “stooges”, hired by the experimenter, also gave one clear but false answer (Asch, 1951). In this experiment people had to judge which line was the same length as three comparison lines. In the context of the covid-19 crisis, individuals with doubts about the lockdown may be less likely to voice them when faced with a social circle who outwardly supports the measures. The public narrative in support of the lockdown may make people reluctant to raise differing opinions, rather choosing to conform to society as a whole, and their own social circles (cf. Whiten, 2019). In the Milgram obedience experiment, it was shown that people were prepared to potentially harm another person by giving an electric shock to a “learner”. This experiment showed that ordinary people could be persuaded to harm other people, if an authority figure asked this, in this case, giving gradually higher shocks, that gradually went up the more “wrong” answers a student gave (Milgram, 1963). Two-thirds of the participants continued to the highest level of volts, which were potentially deadly. These experiments showed the majority of ordinary people are prepared to follow orders given by an authority figure, even if it involved killing innocent human beings. The Stanford Prison experiments sought to find out if ordinary students were randomly assigned to play guard or prisoner as social roles, and concluded that people are willing to do so, especially in stereotyped roles. These experiments have been repeated many times and influence research even today (Haslam and Reicher, 2017), even though the Stanford Prison experiment had some fatal flaws in design and carrying out of the experiment (le Texier, 2019). In general, the studies show that conformity and obedience are very common, and people have an innate tendency to follow the group and/or a leader (Cialdini and Goldstein, 2004).

Although in many instances conformity and obedience are functional, in these experiments and in some instances in real life, conformity and obedience can become dysfunctional and even harmful. A review by Cialdini and Goldstein (2004, p. 61) argued that people are in general “motivated to form accurate perceptions of reality and react accordingly, to develop and preserve meaningful social relationships, and to maintain a favorable self-concept.” In general, compared to conformity, obedience seems to induce greater cognitive conflict (Xie et al., 2016). Social influence theory postulates that attitudes, beliefs and action are influenced through the processes of compliance, identification and internalization (Kelman, 1958). This concerns not only behaviors that are asked from the general public by the government, such as social distancing, but also for instance cases where family members are denied access to a dying parent in a care home. As many of the measures are not evidence-based, the public could have demanded proof for the extent to which the measures were evidence-based and proportional and/or opt for civil disobedience (cf. Ioannidis, 2020). However, because many psychological tactics (Andrews et al., 2020; Bavel et al., 2020), along with agnotology and rules and regulations have been used to influence behavior (cf. Cohen and Kupferschmidt, 2020). The extent to which people do conform and go along with the measures, this will enhance the negative side effects. Unfortunately, both fear of Covid-19 itself as well as the negative side effects of the measures may cause high levels of stress and anxiety, and in turn a compromised immune system. This will be described below.

## Paradoxical Effects of the Lockdown: Stress and Anxiety Caused by Framing and Lockdown Measures Negatively Impact the Immune System and Health Outcomes

Stress occurs often when people face challenging or difficult situations (i.e., stressors) resulting in physiological and psychological responses (stress responses). One of bodily systems reacting to these stressors is our immune system. In acute stress the body reacts to stress with the increase of pro-inflammatory cytokines. The body is prepared for a fight or flight response. Acute stress in a healthy human is quite harmless, but stress that last for days, weeks, months or years can be harmful (Azza et al., 2019). It can result in a state of chronic systemic inflammation which in turn results in the development of chronic diseases. For example, it is well known that chronic stress increase susceptibility to some types of cancer by suppressing Type 1 cytokines and protective T-cells. Chronic stress exacerbates all kinds of pathological immune responses, resulting is diseases and premature death (Dhabhar, 2014). Especially people with prior childhood trauma may be at risk (Azza et al., 2019). As people age, they face a significant lower ability to face stressors with an appropriate immune response. This includes physical stress, but also psychological stress (Morey et al., 2015; Prenderville et al., 2015). In the current situation, the framing of the situation and lockdown measures create stress and anxiety due to a variety of causes (See Figure 1). Furthermore, the stress associated with this and the massive number of job losses also translates into a shorter life span (Roelfs et al., 2011), as stress is involved in the development, maintenance, or exacerbation of many mental and physical health conditions and is also related to accelerated biological aging and premature mortality (Slavich, 2016). So while a lockdown on a small scale may make sense (a small number of people in quarantine, their health and immune system gets compromised) are we now doing this for both the people we aim to protect as well as the people that are expected to have relatively mild symptoms once infected (healthy young people). Paradoxically then, the measures aimed at protecting the vulnerable, compromise the immune systems of both healthy young people, as well as vulnerable people, such as older people with one or more underlying diseases. Many countries have chosen to put vulnerable elderly people in complete social and physical isolation from their relatives and from society, in the hope to protect them from infection and so saving their lives and preventing death. But this forced social and physical isolation is a serious stressor with well-known detrimental effects on physical and psychological health (Brooks et al., 2020). Chronic stress in advanced age will accelerate aging and dysfunction of the immune system. Chronic stress shortens our telomeres and the shortening of telomeres is linked with all kind of diseases and death (Holt-Lunstad et al., 2010). It is found that the influence of the social relationships on these factors is comparable with well-established risk factors as smoking and arterial hypertension (Holt-Lunstad et al., 2010).

The duration of the social and physical isolation is of importance. During the SARS outbreak people that were isolated for more than 10 days showed significantly higher post-traumatic stress syndrome than those who were isolated less than 10 days (Hawryluck et al., 2004). In many countries under present corona lockdown elderly people are isolated up to a few months. Social and physical isolation is commonly associated with loneliness. This is especially the case in forced isolation in old age (for a meta-analysis see Holt-Lunstad et al., 2015) where loneliness is strongly associated with increased mortality (Eng et al., 2002; Giles et al., 2005; Pantell et al., 2013). In contrast, a study by Cohen et al. (1997) concluded that having more diverse social networks is associated with a greater resistance to upper respiratory illness. So depriving people from their liberty and normal psycho-social interactions in the need to prevent infection and death and for the good of the society is contentious. Paradoxically, instead of preventing disease and death it can also induce disease and death. Therefore, it is important to know how people can cope with the current situation. Some of the negative side effects can be moderated by the coping styles, ranging from functional to dysfunctional (Veer et al., 2020).

## Coping Styles Can Alleviate or Exacerbate Some of the Side Effects

Although the Covid-19 outbreak has caused a tremendous amount of stress on the general population (Zhang et al., 2020), prior research has identified stable psychological traits, and several circumstances that predict perceived stress under these circumstances (Flesia et al., 2020). People can react to prolonged stress with coping, which can range from functional, such as a healthy lifestyle and seeking support to more dysfunctional, such as withdrawal and substance use (See Figure 1). The negative effects of stress related outcomes can (in part) be counteracted by functional coping styles (Yu et al., 2020). Functional coping styles and several interventions have been related to better resilience, emotion regulation and health outcomes (Santarnecchi et al., 2018; Ho et al., 2020; Polizzi et al., 2020). These strategies can diminish the effects and over time (in part) counteract the negative consequences of the lockdown. Unfortunately, the lockdowns and related increase in anxiety, depression, and PTSD (Guessoum et al., 2020), and as many sports facilities were closed this related to changes in life style such as eating more, and sporting less (Di Renzo et al., 2020; Górnicka et al., 2020; Pellegrini et al., 2020). Paradoxically, *functional coping styles* in order to offset some of these negative effects have been blocked in some ways due to the measures and this may have led to downward spirals in terms of (mental) health (Ibrahimagić et al., 2020). These include: a healthy lifestyle, such as eating healthy, seeking support and relaxing exercises. Unfortunately, due to the lockdowns, many people have starting snacking more and gained weight (Di Renzo et al., 2020), as well as reduced daily physical activity, even though the practice of physically active lifestyles is recommended to counteract (mental) health consequences of the lockdowns and COVID-19 pandemic (Lim and Pranata, 2020). Seeking social support, while helpful in reducing stress, was also harder, as people experienced social isolation. Relaxing exercises could have been done at home, but the question is how much these were done by people to relieve stress. On the other hand, people may try to make sense of the situation and may seek out other sources of information than the ones readily presented to them. The central aim of science is to make sense of the world, and systematic and focused scientific sense-making may help people understand better what is going on. In that sense, it could be quite functional (Passmore et al., 2014). This may at the same time help fight the negative effect of agnotology induced doubt and confusion.

### Dysfunctional Coping Strategies

Dysfunctional coping strategies, such as withdrawal/ruminating, substance use, taking tranquilizers and excessive gaming can exacerbate the negative effects of the lockdown measures (cf. Wang H. et al., 2020), and it seems that another paradox is created by the fact that the people experiencing a higher level of psychological distress, also had more dysfunctional coping styles (Wang H. et al., 2020). In turn, people with substance use disorder, have a higher risk of contracting Covid-19, and the increase in substance use may be observed for years after the lockdown (Mallet et al., 2020). In going forward, it is important to try to make sure that this group of people adopts more functional coping styles (Yu et al., 2020; Zhang et al., 2020).

### Sense Making

Sense making may be a third, hitherto unexplored way of coping. In uncertain times like these, people may try to cope by making sense of the situation (e.g., Stephens et al., 2020). Scientific sense making in terms of trying to make sense of what is going on could be quite functional (Passmore et al., 2014). However, in uncertain time superstitious beliefs, conspiracy theories, and cognitive dissonance reduction represent ways in which people try to make sense and cognize an ambiguous situation that seems beyond comprehension. As many people are forced by governments into behaviors they would normally not adhere to, cognitive dissonance and superstitious beliefs can also explain why people will persist in certain behaviors, even when it becomes known that the majority of these are not helpful or evidence-based (Ioannidis, 2020). In general, people strive for consistency between cognition and behavior, and have a need to see a relation between behavior and outcomes, even if this relation is not there (Tsang, 2004). For instance, people may maintain behaviors, even after some lockdown measures have been lifted and for instance call in sick for work out of fear to become infected. Moreover, many people will think that the more sacrifices they make, the more helpful it must be (cf. Elliot and Devine, 1994). Also governments may believe they need to take decisive action and may resort to non-evidence based lockdown measures that do more harm than good (Ioannidis, 2020), and adhering to those may represent a form of superstitious bias that action is better than non-action (cf. Schippers et al., 2014), and the relation between the behavior and outcome is spurious, or not as strong as one believes (Schippers and van Lange, 2006). Superstition is widespread in most human societies, even today (Tsang, 2004; Vyse, 2013). Especially in times of uncertainty, there is a need for humans to rely on superstitious behaviors and/or beliefs (Schippers and van Lange, 2006). These beliefs are held by many people, also people we regard as intelligent (for a review see Brooks et al., 2016). Prior research has shown that superstitious beliefs and behaviors can reduce uncertainty-induced anxiety (Schippers and van Lange, 2006; Brooks et al., 2016). In the case of today’s uncertainty, where the stakes are high, and mortality salience is excessively heightened by the constant media coverage of the number of deaths as a result of Covid-19, as well as uncertainty about just how contagious and deadly the virus is, governments and individuals alike will resort to superstitious beliefs and behaviors in order to reduce anxiety. Although most definitions have some element of the belief in magic as part of the definition, early research suggests that merely seeing a connection between an action and an outcome that is not really there is also a form of superstition (Skinner, 1948). Acting on it, this performing rituals as ways to reduce anxiety, is referred to as superstitious rituals (Schippers and van Lange, 2006; Brooks et al., 2016). Although this is a form of bias, recent research suggests that oftentimes, even though people recognize it as a form of superstition, they choose to hold on to it “just in case”. This suggests that even if people detect the error, and may admit that this is a form of superstition, they may choose not to correct it. This process has been referred to as acquiescence (Brooks et al., 2016). The behaviors asked from people are in part superstitious, and may have an adaptive function (Markle, 2010), but also have relations with obsessive-compulsive behavior (OCD). As not all behaviors are necessary (e.g., staying indoors when healthy; (Born et al., 2020), some of these are more OCD like and superstitious (Moulding and Kyrios, 2006; Spears, 2014). Although people have various behaviors to counteract stress and possibly exert control over situations (Moulding and Kyrios, 2006), many people still experience mounting stress, not only by the threat of the virus, but also by the way the situation is framed, as well as the effect of the lockdown itself. This type of framing helps in sustaining the behavior, sometimes even when disconfirming information is presented (Russell and Jones, 1980). Even so, and even though people are confronted with conflicting information, this adds to the stress and anxiety they are seeking to reduce. At the same time, many people feel that there are too many uncertainties in current situation to be able to conclude what is the ‘right’ way of acting, even though it becomes clear that the ripple effects of the current action are quite severe in the long run (Zetzsche, 2020).

Cognitive dissonance will create tension between the belief that the sacrifices people make are necessary and the belief that some of these behaviors may be causing more harm than good in terms of mental health (McGrath, 2017). The unpleasant tension stemming from conflicting beliefs then leads people to decide that the lockdown must be useful, and people also try to get doubters to reconsider their position, even in the face of clear evidence of overwhelming negative side effects. Ironically, the term “cognitive dissonance” is based on research into a religious sect that believed the world would end (Festinger, 1957). They sold all their belongings and waited for a flying saucer to come and pick them up. When that subsequently (of course) didn’t happen, that was no reason to change their beliefs. They now stated that they had saved the world and that God had decided to spare it due to their actions. In this way, they did not have to adjust their core beliefs, instead changing their view of the facts to fit into their existing narrative (Festinger, 1957). This may also happen, as people believe there is a strong relation between performing behaviors recommended (e.g., social and physical distancing, and forced isolation) and they see that it works, as the spread of the disease seem to be contained. However, several studies have indicated that the disease may play itself out after a certain period of time, independent of the measures taken (Ben-Israel, 2020; Ederer, 2020). Also, people seek for an explanation, and they feel the need to explain large events with proportionally large causes (Leman and Cinnirella, 2007), and as they note that the side-effects of the response to Covid-19 are quite severe, many resort to conspiracy theories (Smallman, 2015; Bavel et al., 2020). Unfortunately, although it may be related to decreased anxiety, conspiracy theories are in general more appealing than satisfying (Douglas et al., 2017). Prior work has found that a lack of control increases conspiracy thoughts and superstitious beliefs (Huang and Whitson, 2020).

Coping strategies such as cognitive dissonance reduction, superstitious beliefs and rituals, as well sense making through conspiracy theories, although somewhat functional in terms of reducing anxiety, are not satisfying key psychological needs in the long run (cf. Douglas et al., 2017) Nevertheless, in the short run, stress and anxiety are high and people are motivated to reduce these emotions, via a variety of behaviors and coping mechanisms (See Figure 1).

## Accelerating Functional Coping Processes Through Life Crafting

The current crisis has increased the need for functional coping with traumatic experiences and negative emotions. While many people experience a downward trend in terms of emotions, depression and trauma, broaden-and-built theory offers insights in how to reverse those processes (Fredrickson, 2001). As it will be hard to stop the negative side effects, such as job loss and prolonged fear, from playing out, it is imperative to at least try to minimize the negative mental health effects. Broaden-and-built theory postulates that thinking about an idealized future will be associated with positive thought about that future. The theory and findings suggest that the capacity to experience positive emotions is related to the human capacity to bounce back from negative experiences and is related to human flourishing (Fredrickson, 2001). Specifically, functional coping processes and the experience of positive emotions can be accelerated by a process of expressive writing about one’s ideal life (for reviews see Schippers and Ziegler, 2019; de Jong et al., 2020). Prior research in a student population has shown that a brief, 4–6 h written and staged goal-setting intervention, that includes both writing about ideal life and goals, with goal achievement plans, improved academic performance (Morisano et al., 2010; Schippers et al., 2020), and has been shown to close the gender and ethnic minority achievement gap (Schippers et al., 2015). As it seems that for many people their purpose in life needs to be redefined, for instance as a result of job loss, life crafting offers a way to find (renewed) purpose and meaning (Schippers and Ziegler, 2019; de Jong et al., 2020). Meaning in life has been associated with numerous positive physical and mental health outcomes, such as (mental) health, adaptive coping, and decreased mortality (Heintzelman et al., 2013), and is a protective mechanism against mortality salience and existential anxiety (for a meta-analysis see Burke et al., 2010). Life crafting is based on techniques that originally were designed for expressive writing about emotional and traumatic experiences (Pennebaker, 1997) and coping processes (Pennebaker et al., 1990). This work showed that writing about emotional experiences is related to significant physical and mental health improvements (Pennebaker, 1997). A variation of the writing paradigm, writing about the best possible future self, was both less upsetting than writing about trauma, but had similar effects in terms of significant increased subjective well-being (King, 2001). Even 2-min writing exercise for 2 days showed reduced health complaints at follow-up (Burton and King, 2008). The life crafting intervention has three main elements: (1) discovering values and passion (2) writing about goals and goal achievement plans, and (3) public commitment to goals. During the writing exercise, people write about what they like to do, competencies they would like to acquire, relationships at home, work and in leisure time, possible future career, as well as their ideal versus less ideal imagined future. On the basis of this, people formulate concrete goals, order these in terms of importance and write detailed plans including goal monitoring and “if-then” plans. The third part then is about making a photo with a statement communicating their goals to the world, be it friends, or co-workers (for reviews see Schippers and Ziegler, 2019; de Jong et al., 2020). Using this online intervention, I hypothesize that people will experience accelerated functional coping and this may serve as a way to restore well-being.

## Discussion

The current review focused on the psychological and behavioral consequences of the lockdown and suggested that the negative effects are serious and may very well outweigh the possible positive effects of the lockdown for the general population (Izaguirre-Torres and Siche, 2020). As Brooks et al. (2020, p. 919) noted: “……there can be long-term consequences that affect not just the people quarantined but also the health-care system that administered the quarantine and the politicians and public health officials who mandated it.” Indeed, the measures create a paradoxical situation, where not only people getting ill are negatively affected, but also the healthy people in the lockdown situation (Liang et al., 2020; Zhang et al., 2020). The current paper falls necessarily short in listing all negative side and ripple effects, because (a) the situation is still unfolding, and (b) many of these effects are still unknown or (c) could be counteracted if governments make this a priority. The current paradoxical situation, could be addressed by (1) evidence-based optimized decision making by governments (2) making use of information and scientific findings in an unbiased manner (3) stating clear goals for what we are trying to achieve with the measures and (4) an evidence-based way of public health measures that avoid or counteract the negative side effects (Horesh and Brown, 2020). Medium and longer term planning is needed to rebuild the economy as well as a mental health care system aimed at reversing the side effects of the measures. As several studies have suggested ways forward from here in terms of the economic impact (Boone et al., 2020; Zetzsche, 2020), as well as ways to boost the human immune system in order to prevent people from getting sick (Nilashi et al., 2020; Taghizadeh-Hesary and Akbari, 2020). In order to make sure that some of the negative mental health effects are counteracted, this calls for effective evidence-based interventions (Wilson, 2011; Figueroa and Aguilera, 2020), that can be made available online and are scalable (Schippers and Ziegler, 2019; de Jong et al., 2020). Although tele-health and video consultation can alleviate the immediate problems associated with the lock-down, (Barsom et al., 2020; Zhou et al., 2020), there may not be enough staff to effectively treat all people that will need mental health care in the aftermath of the global lockdown (Figueroa and Aguilera, 2020; Torales et al., 2020). Next to giving the public more information about effective coping styles (Ibrahimagić et al., 2020), an interesting avenue is to make writing interventions available to the wider public, that have proven to have many (mental) health benefits (Lepore and Smyth, 2002; Schippers et al., 2015), as well as performance benefits (Schippers and Ziegler, 2019; de Jong et al., 2020; Schippers et al., 2020). This type of care could even be delivered by a life crafting chatbot (Dekker et al., 2020). Life crafting, or the process of reflecting and writing about present and ideal future life, also including making plans and changes accordingly, can help to restore and improve both meaning in life and psychological and physical health (Schippers and Ziegler, 2019). This may be now more needed than ever (de Jong et al., 2020; Figueroa and Aguilera, 2020). Digital mental health tools are a way forward in counteracting the negative mental health effects in the wake of the Covid-19 crisis and investing in making these available for large groups of people in need is key (Figueroa and Aguilera, 2020). As we are arguably facing the largest humanitarian disaster in the history of mankind, caused by the lockdown measures, it is my hope that the negative side effects will, to some extent, be counteracted via smart interventions and community care.

## Author Contributions

MS played the primary role in the conceptual conception of the manuscript, and wrote, reviewed, and revised the manuscript.

## Conflict of Interest

The author declares that the research was conducted in the absence of any commercial or financial relationships that could be construed as a potential conflict of interest.

## Footnotes

1 The virus at the center of this crisis is called SARS-CoV-2, while the disease caused by this virus is COVID-19. As many authors have started using the term ‘COVID-19 crisis’, we will use it throughout this paper to refer to the crisis for the sake of simplicity, and readability.
